# Large‐Aperture, WFOV Broadband Achromatic Metalens Enabled by Forward Mapping Optimization and Hybrid Algorithmic Framework

**DOI:** 10.1002/nap2.70228

**Published:** 2026-08-06

**Authors:** Zongyuan Chen, Wangzhe Zhou, Shaoqi Li, Yiyi Li, Nan Gu, Man Yuan, Junbo Yang

**Affiliations:** ^1^ College of Science National University of Defense Technology Changsha China

**Keywords:** broadband achromatic, forward mapping optimization, large aperture, metalens, wide field of view

## Abstract

Achieving large aperture, wide field of view, and broadband achromatic imaging in the visible represents a critical challenge for advancing metalens toward practical applications. This work demonstrates a visible‐spectrum metalens that simultaneously realizes a 4.584 mm aperture, a 100° field of view, and achromatic imaging over 400–680 nm. This study develops a forward mapping optimization (FMO) to overcome the dual bottlenecks of computational complexity and the need for global balance in aberration correction encountered by traditional point‐by‐point compensation approaches. This reduces computational complexity from O(*N* ^2^) to O(*N*). Simultaneously, a hybrid algorithmic framework is employed to significantly leverage the advantages of both global and local optimization algorithms. Simulation results demonstrate that this metalens achieves excellent achromatic imaging, with a maximum focal length deviation of less than 0.3%. The proposed approach features very low computational cost, requiring only a single 6‐core, 12‐thread computer. This method also exhibits rapid convergence and high computational precision. This research can be extended to other similar designs, laying the foundation for applications in novel smartphone cameras, robotic vision systems, VR/AR, and medical endoscopic imaging.

## Graphical abstract

A novel large aperture metalens design strategy achieves broadband achromatic imaging across the visible spectrum (400–680 nm) with a 100° field of view. By incorporating forward mapping optimization and a hybrid algorithm framework, this approach overcomes the limit between aperture size, field of view, and chromatic aberration correction, providing a reference for designing compact imaging systems.

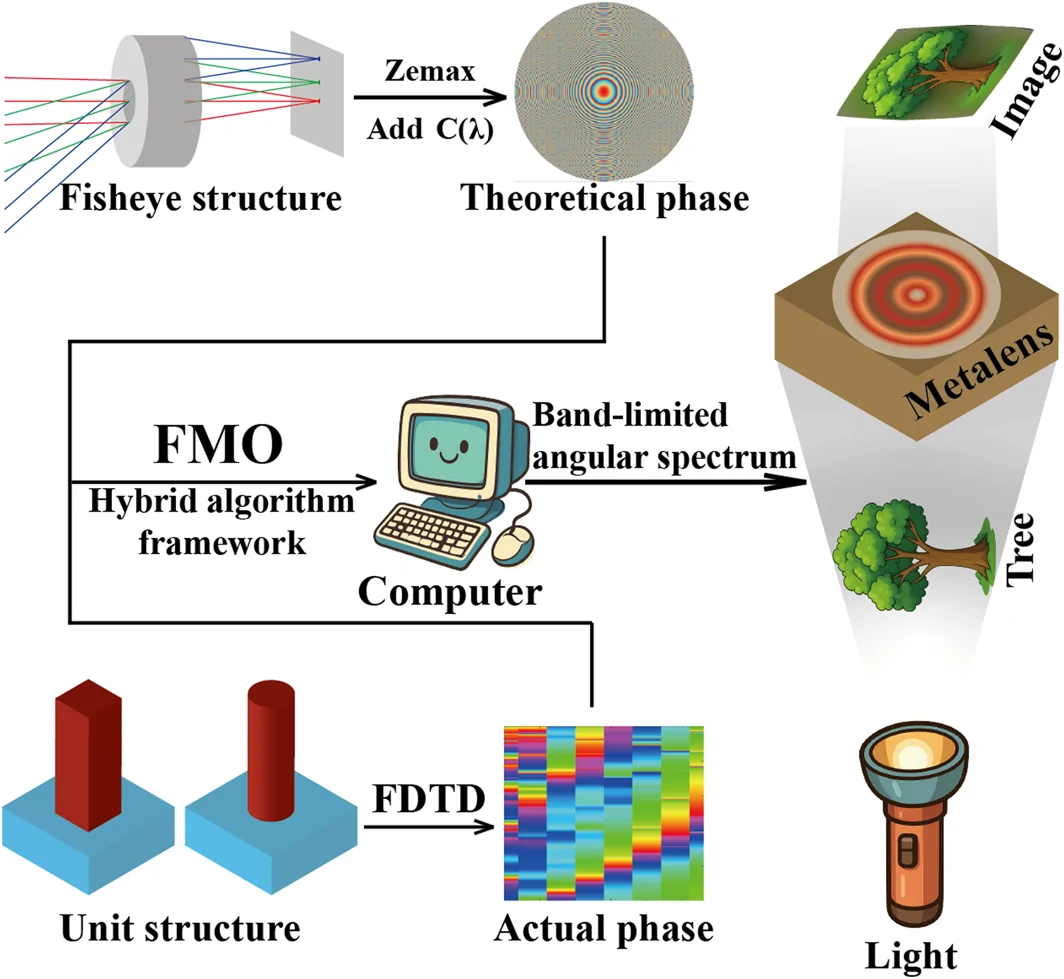

## 1 Introduction

The evolution of optical imaging technology has consistently revolved around achieving more precise manipulation of light wavefronts. Building upon this foundation, each breakthrough in technological paradigms has propelled imaging systems toward greater compactness, efficiency, and intelligence.

Metasurfaces, two‐dimensional optical materials composed of subwavelength‐scale artificial atoms arranged in specific sequences [1], have fundamentally reshaped people's understanding of the boundaries in light‐field manipulation in recent years. Unlike the physical mechanisms of traditional optical elements, which rely on accumulated optical paths and macroscopic surface curvature for wavefront control, metasurfaces achieve precise manipulation of multiple degrees of freedom, including amplitude, phase, polarization, and frequency, within extremely thin structures through specially designed nanoscale units [2, 3, 4, 5, 6]. This revolutionary light‐field manipulation capability enables the development of ultra‐compact, planar, and multifunctional integrated optical systems, paving the way for a new technological path toward miniaturization and intelligent operation in next‐generation photonic devices [7, 8, 9, 10, 11].

Among the numerous groundbreaking applications of metasurfaces, metalenses stand out as an ideal replacement for traditional refractive and reflective lenses, demonstrating exceptionally broad application prospects. Theoretically, a single metalens element can achieve the imaging capabilities of multiple complex lens assemblies. This revolutionary advantage grants it immense application potential in fields demanding stringent size, weight, and integration constraints—such as mobile device camera modules, medical endoscopes, augmented/virtual reality (AR/VR) displays, and lightweight drone vision systems [7, 8, 12, 13, 14, 15, 16]. Particularly in the visible light spectrum (400–700 nm)—the core wavelength range for human visual perception and the operation of most imaging systems—the development of high‐performance metalens is regarded as a pivotal milestone in advancing this technology from the laboratory to industrial applications [17, 18].

However, advancing metalens from laboratory demonstrations to practical applications requires simultaneously overcoming three core challenges, which inherently form an interdependent performance triangle [19]: broadband achromatic correction, large aperture (or high numerical aperture), and wide field of view (WFOV). These performance metrics are physically interdependent: wide band achromaticity is a fundamental requirement for visible light imaging. The phase modulation capability of metalens stems from the electromagnetic resonance properties of their nanostructures, which are inherently highly wavelength‐dependent [20, 21]. This results in severe chromatic aberration in traditional metalens based on single structure designs, causing significant shifts in the focal positions of light at different wavelengths and severely degrading imaging quality [22]. Researchers have developed various chromatic aberration correction strategies, such as utilizing layered structures to independently control phase and dispersion at different depths [23], or combining groups of nanostructures with distinct dispersion characteristics within a single interface [24]. However, these approaches often face significant challenges in terms of design complexity and fabrication tolerances. Large apertures are a prerequisite for achieving high resolution imaging. However, for every doubling of the aperture of a metalens, the number of independently optimized nanoscale units required increases quadratically. The computational complexity of traditional design methods is typically O(*N* ^2^), making it extremely challenging to design metalens with millimeter scale or larger apertures in the visible [25, 26, 27]. This severely limits their application in high resolution imaging. A large field of view is an essential characteristic for most imaging systems. Traditional metalens achieve perfect focusing only under the paraxial approximation. When light is incident off axis, severe aberrations occur, such as coma, astigmatism, and field curvature [28]. Although introducing an asymmetric phase term can extend the field of view to some extent [29], this approach requires simultaneous optimization of the response characteristics for each nanostructure at multiple incident angles, significantly increasing the design complexity. Especially in ultra‐wide fields of view (> 60°), wavefront aberrations become extremely complex, making it difficult for traditional design methods to achieve effective global optimization. Furthermore, expanding the field of view requires precise wavefront modulation to flatten the phase profile, which conflicts with the rapid phase changes needed to achieve large apertures. This makes it challenging to simultaneously optimize the response of tens of millions of nanoscale units across multiple wavelengths and angles [30, 31, 32, 33].

Shalaginov et al. employed a fisheye lens structure to expand the field of view of a metalens, achieving a record‐breaking diffraction‐limited field of view of 170° [34]; Martins et al. designed a quadratic phase metalens, achieving a WFOV metalens at 532 nm wavelength [35]; Yang et al. employed deep learning algorithms to design an achromatic metalens with a 60° field of view [32]; Huang et al. developed a reverse design and deep learning approach, significantly reducing chromatic aberration, while also observing noticeable degradation in image quality along the periphery [36].

Despite recent significant advances in broadband achromatic correction or WFOV performance within the visible, integrating large aperture (> 1 mm), broadband achromatic correction, and WFOV into a single device has proven challenging. The fundamental bottleneck lies in the absence of a design framework capable of globally and efficiently coordinating multiple physical objectives (multiple wavelengths, multiple angles) while simultaneously reducing computational complexity.

Table 1 presents existing works in this field. As shown in the table, very few studies have achieved all three of the aforementioned objectives while maintaining excellent performance; generally, researchers can only optimize two of the three dimensions, which poses certain challenges for comparing our work with existing studies. Shalaginov et al. [37] achieved a large 170° field of view and 1 mm aperture, but their work was limited to a single wavelength; Huang et al. [42] realized a 5 mm aperture and continuous‐wave achromaticity, yet their field of view was only 40°. Considering that other methods focus on optimizing single or dual variables, this study reduces computational cost while maintaining multi‐objective optimization capabilities, demonstrating the advantage of a comprehensive, holistic approach.

**TABLE 1:** Current related work on this study.

Work	Aperture	Wavelength band	FOV [°]	Design methods/structure
Shalaginov et al. [37]	1 mm	5.2 μm	> 170	Fisheye
Groever et al. [38]	NA	470–660 nm	50	Doublet
Pu et al. [39]	40 μm	490, 532, 632.8 nm	> 160	Quadratic metalens
Lin et al. [40]	NA	650–1000 nm	NA	Meta‐atoms on a SPDC layer
Richards et al. [41]	90 μm	400–700 nm	NA	Hybrid microlens
Fan et al. [30]	110 μm	532 nm	> 170	Hexagonal arrangement, fisheye
Huang et al. [42]	5 mm	8–12 μm	40	Binary phase metalens
Yang et al. [32]	200 μm	1.0–1.5 μm	180	Deep learning
Sun et al. [43]	450 + 240 μm	470 nm	27	Annular illumination, doublet
Luo et al. [31]	38 μm	492–592 nm	40	Fisheye
This work	4.584 mm	400–680 nm	100	Fisheye, FMO

We have devised a novel design paradigm called forward mapping optimization (FMO). The core of the FMO approach lies in leveraging the classifiability of metalens array configurations to bypass traditional point‐by‐point optimization, significantly reducing the computational complexity of the design process from O(*N* ^2^) to O(*N*). Using this approach, we successfully design and validate a 4.584 mm diameter metalens that demonstrates outstanding achromatic performance, wide‐field imaging capabilities across the 400–680 nm visible spectrum and a 100° full field of view, and excellent focusing characteristics. Our work not only demonstrates a high‐performance device, but more importantly, provides a scalable design framework that opens new avenues for the rapid development of high‐performance, multifunctional metadevices.

## 2 Design Method

### 2.1 Fundamentals and Algorithm Design

For normal incidence, the traditional phase formula for a focusing metalens can be expressed as Equation (1) [44].

(1) $$\varphi \left(r,\lambda \right)=-\frac{2\pi }{\lambda }\left(\sqrt{r^{2}+f^{2}}-f\right),$$

here λ is the wavelength, r is the distance from a point on the surface of the metalens to the center of the metalens, and f is the focal length of the metalens. At this point, the phase distribution of the metalens at a single wavelength depends only on r and f.

When the light source is incident off‐axis, the phase distribution of the focusing metalens must also account for the angle of incidence. In this case, the phase formula can be expressed as Equation (2) [45].

(2) $$\varphi \left(x,y,\alpha ,\lambda \right)=-\frac{2\pi }{\lambda }\left[x\;\sin\;\alpha_{x}+y\;\sin\;\alpha_{y}+\sqrt{f^{2}+{\left(x-x_{0}\right)}^{2}+{\left(y-y_{0}\right)}^{2}}-\sqrt{f^{2}+{x_{0}}^{2}+{y_{0}}^{2}}\right],$$

where $\alpha_{x}$ and $\alpha_{y}$ represent the angles between the incident angle and the xy axes, and $x_{0}$ and$y_{0}$ denote the horizontal and vertical coordinates of the focal point. This equation involves multiple interrelated variables that require simultaneous optimization. However, in practical applications, it is challenging to simultaneously satisfy all response requirements corresponding to the unit structure. Therefore, it is necessary to identify a balance point among these variables to ensure the final response best meets the design specifications.

To meet the requirements of wide field metalens design, the surface phase must exhibit good symmetry to ensure low off‐axis aberrations across a wide field of view [31]. Therefore, in practical design, we adopt the fisheye configuration commonly used in traditional wide field lenses, which allows the conversion of incident angle parameters into lens structure parameters. Building upon this foundation, we first transform the $x_{0}$ and$y_{0}$ parameters in the phase formula into polar coordinates. Subsequently, we perform a polynomial expansion while preserving symmetry, yielding a function expressed as Equation (3) [31].

(3) $$\varphi \left(r\right)=\sum_{i}^{n=6}A_{i}{\left(\frac{r}{R}\right)}^{2i},$$

here $A_{i}$ represents the polynomial expansion coefficient, R denotes the normalized radius, and n is the number of expansion terms, which we set to 6, ensuring sufficient precision while minimizing computational cost. At this point, φ(r) becomes a function solely dependent on r, with distinct expansion coefficients corresponding to different wavelengths. We employ commercial optical design software to simulate multi‐angle light incidence (maximum incidence angle of 50°), optimizing the physical lens structure through ray tracing while calculating the polynomial expansion coefficients.

To compensate for chromatic aberration caused by different wavelengths, we introduce a constant parameter factor C(λ) that depends solely on wavelength. The overall phase can then be expressed as Equation (4) [31].

(4) $$\varphi \left(r,\lambda_{j}\right)=\sum_{i}^{n=6}A_{i}{\left(\frac{r}{R}\right)}^{2i}+C\left(\lambda_{j}\right),$$

here j represents the number of selected wavelengths. To better compensate for chromatic aberration across the entire broadband range, we set j to 8.

We preliminarily estimate that the number of units requiring optimization for the designed metalens will be on the order of 10^8^, far exceeding the computational power of current conventional computers. To address this, we design a hybrid algorithm optimization framework combining Bayesian optimization as the primary method with particle swarm optimization (PSO) as the post‐processing step: Bayesian optimization rapidly identifies potential regions within the vast parameter space, followed by PSO performing local optimization within several identified potential regions. This framework is not limited to the algorithms mentioned; other types of algorithms can also be implemented. The specific convergence properties are presented in Supporting Information S1: Section S1. In reality, relying solely on the hybrid optimization framework only enhances the efficiency of finding solutions within the determined parameter space. Further reduction of the parameter space's size is necessary to enable computations to proceed on conventional computers.

To this end, based on the principle of symmetry, we have devised a novel design paradigm called forward mapping optimization (FMO). First, the metalens is divided into four symmetrical sections with the center point as the origin. The distances between the centers of all grid cells in the first quadrant and the origin are defined as set *A*. Next, a reference cell set *B* is established, containing *N* cells, ensuring that all cells within this set exhibit unique distribution characteristics across the entire metalens. In practice, we select *N* grid cells traversed by a ray originating from the origin. Next, through divisions such as nearest neighbors and second nearest neighbors, all elements in set *A* are mapped to set *B*. Based on this, set *A* is divided into *N* categories. At this point, *N* × *N* grids are mapped onto *N* grids, significantly reducing computational complexity. Finally, the hybrid algorithm performs phase matching optimization for the required phase on the metalens surface within the unit cell library, ultimately yielding the overall structural arrangement. The detailed process is shown in Figure 1.

**FIGURE 1:**
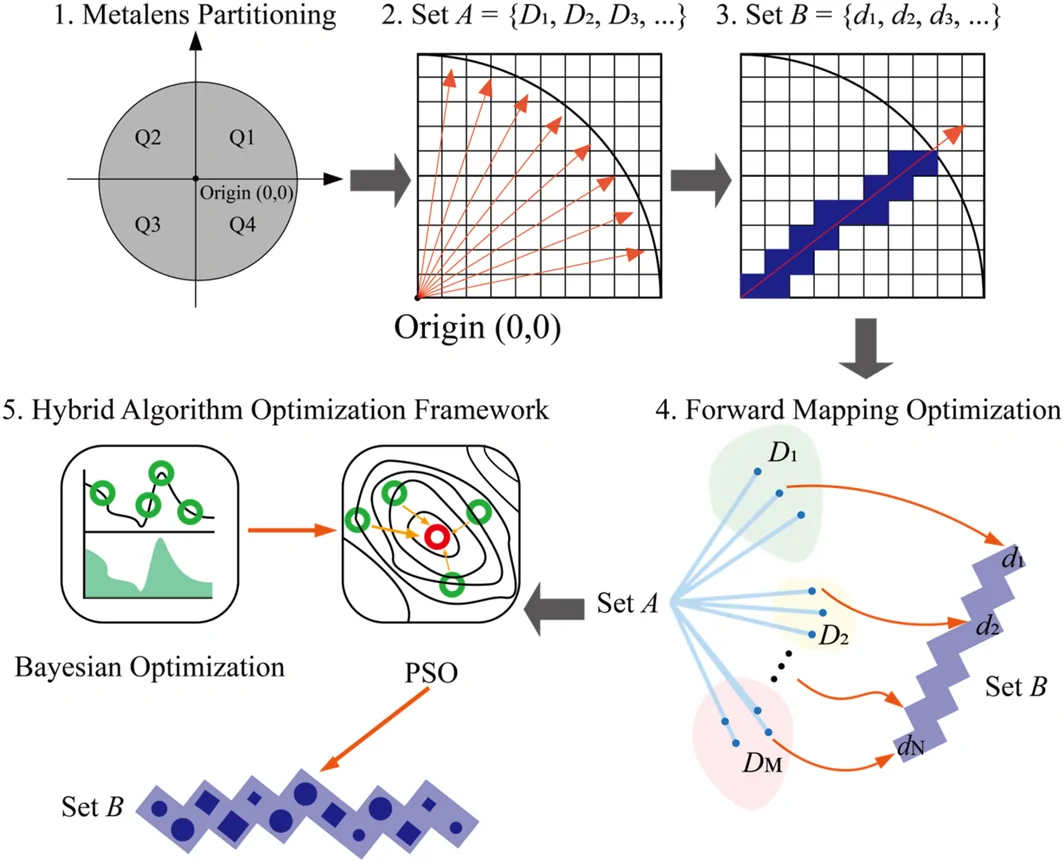
Detailed design process for the FMO and hybrid algorithm framework.

FMO operates within the hybrid algorithm framework, specifically manifested in the formulation of the evaluation function. At this stage, the evaluation function Δφ is defined by Equation (5).

(5) $$\begin{array}{ccc} \Delta \varphi_{j,k} & = & \frac{\left(\varphi \left(r_{k},\lambda_{j}\right)-\varphi_{\text{real}}\left(r_{k},\lambda_{j}\right)\right)\;\mathrm{mod}\;\left(2\pi \right)}{2\pi },\\ \Delta \varphi & = & \sum_{j}\sum_{k}\min\left(\Delta \varphi_{j,k},1-\Delta \varphi_{j,k}\right)\times f_{k}, \end{array}$$

here ${\Delta \varphi }_{j,k}$ denotes the difference between the theoretical phase and the actual phase introduced by the grid k on the metalens at the selected wavelength j, normalized accordingly; $f_{k}$ represents the frequency of element *B* corresponding to grid k across the entire metalens set *A*. Using this algorithm, we map the *N* ^2^ parameter design space onto a set of *N* elements, significantly reducing computational complexity from O(*N* ^2^) to O(*N*). This substantially alleviates computational demands for large aperture metalens design.

### 2.2 Metalens Design

We employ the transmission phase principle, selecting H‐FK95N glass with low dispersion in the visible spectrum as the substrate. The unit cell structure consists of cylindrical and square pillars made of TiO_2_, with a height of 600 nm and a unit cell period of 400 nm, as shown in Figure 2a. The overall metalens configuration, as depicted in Figure 2b, comprises three components: the aperture stop, the substrate, and the metalens. Figure 2c,d shows the surface unit structure simulated distribution of the metalens.

**FIGURE 2:**
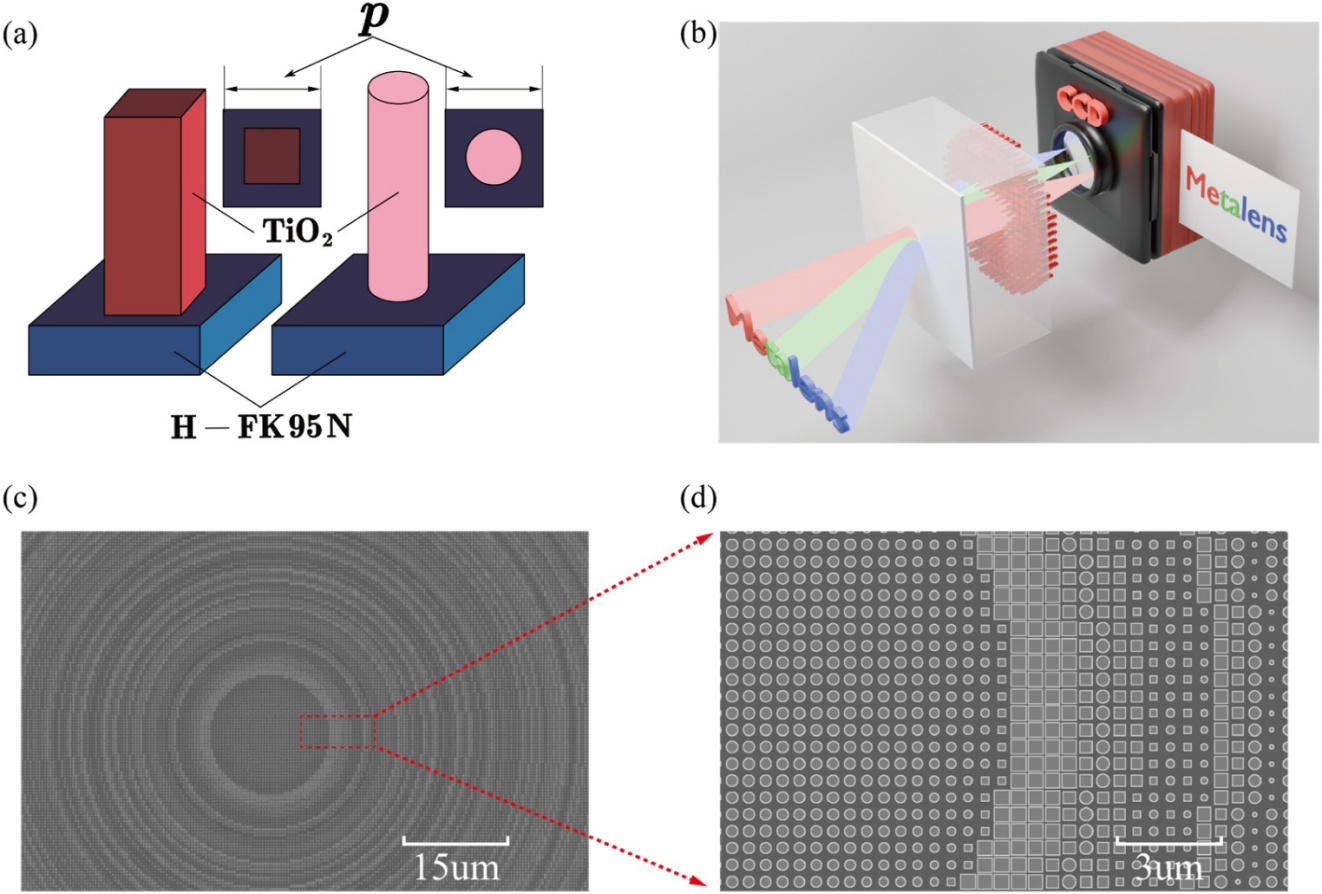
Physical structure and unit cell configuration of metalens. (a) Two unit cell configurations employed, with square and circular cross‐sections, respectively. (b) Fisheye configuration of a wide field metalens, where light incident at different angles focuses at distinct positions on the same focal plane. (c, d) Simulated distribution of metalens surface unit structures.

In the optical simulation software FDTD, the scan parameters are set to vary both the side length and diameter from 100 to 350 nm with a step size of 0.5 nm. Supporting Information S1: Figure S2 displays the results obtained at eight discrete wavelengths during the scan. The scanned parameter library covers a phase range of 0–2π, ensuring superior fitting of surface phase distributions across eight wavelengths during design. To maintain high transmittance, we also scan transmittance profiles (as shown in Supporting Information S1: Figure S2c,d), where selecting cells with high transmittance enhances final imaging quality and focusing efficiency.

To facilitate subsequent processing, we exclude cells with geometric cross‐sectional diameters greater than 300 nm or less than 100 nm when selecting cell structures. This ensures that the minimum distance between any two cells is maintained at 100 nm or more, which aligns with current processing capabilities. Unfortunately, due to the large aperture, the large number of structural units, and the small period, fabricating a metalens of this scale requires a significant amount of time. Furthermore, given the error rate associated with the fabrication of individual structures, when applied to such a large number of surface elements, the estimated yield of the final metalens is expected to be significantly lower. However, this does not exceed the theoretical physical limits, and with the rapid development of this field, technologies for fabricating such metalenses will ensure that our work can be validated in practice.

During iterative optimization in Zemax, we define effective focal length and field of view as the primary design objectives. The field of view (FOV) angle range is set from 0° to 50°, with sampling intervals of 10°, and uniform weighting is applied to ensure balanced imaging performance across the entire FOV range. The objective function is designed primarily to minimize the RMS spot size relative to the center of mass while optimizing the point spread function (PSF) distribution. Through this multi‐wavelength co‐optimization process, a complete set of polynomial phase coefficients is obtained. The phase distribution at 400 nm wavelength obtained through Zemax iterative optimization is shown in Figure 3a, with the corresponding eight sets of coefficients presented in Supporting Information S1: Table S1. Figure 3b shows the standard point pattern diagram for six incident angles under 400 nm incident light. The black circles represent the first Airy disk radius, while the blue indicates the imaging characteristics of light rays on the focal plane. It can be seen that a series of aberrations are inevitably introduced at large angles of incidence, but overall, the system approaches the diffraction limit and exhibits good imaging characteristics.

**FIGURE 3:**
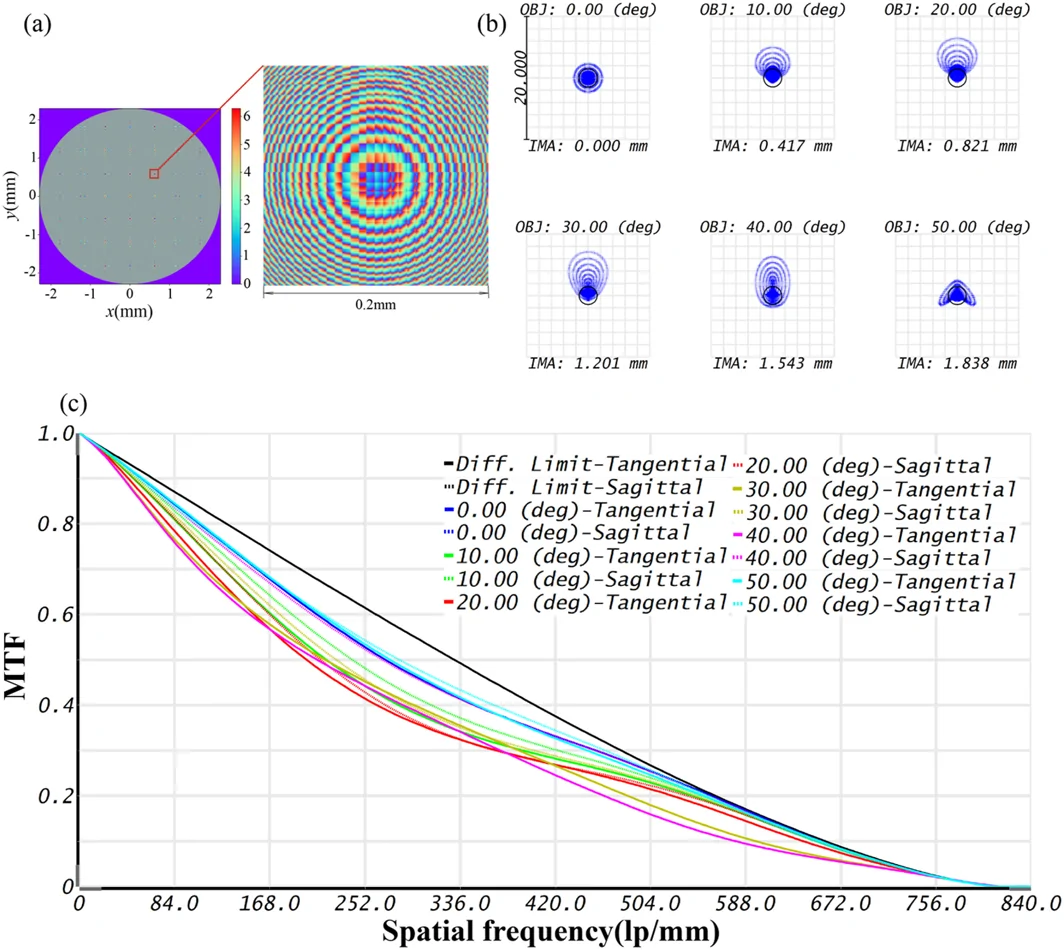
Parameters optimized using Zemax under 400 nm incident light. (a) Planar phase distribution of the metalens. (b) Standard point pattern diagram at different incident angles obtained via ray tracing. (c) MTF curves at various incident angles and diffraction limit.

In optics, the modulation transfer function (MTF) objectively and comprehensively characterizes an imaging system's quality by evaluating its contrast restoration capability. By calculating the diffraction limit (black curve) and plotting MTF curves for multiple incident angles, as shown in Figure 3c, it is evident that the optimized metalens exhibits WFOV imaging performance approaching the diffraction limit. At 276 lp/mm, the MTF curve of the entire system deviates most significantly from the diffraction limit, reaching approximately 0.2; the cutoff frequencies at different angles are comparable to the cutoff frequency at the diffraction limit. Overall, the obtained results satisfy the requirements for a well‐designed system.

Building upon this foundation, we employ the FMO and hybrid algorithm framework to obtain the optimized parameter C(λ) and a series of unit cell parameter distributions.

Using the aforementioned method, we first conduct a verification simulation on a small aperture metalens in FDTD software, specific details are presented in Supporting Information S1: Section S4. The simulation results for this small‐aperture metalens support our next steps.

To summarize the above process, the entire design process is illustrated in Figure 4.

**FIGURE 4:**
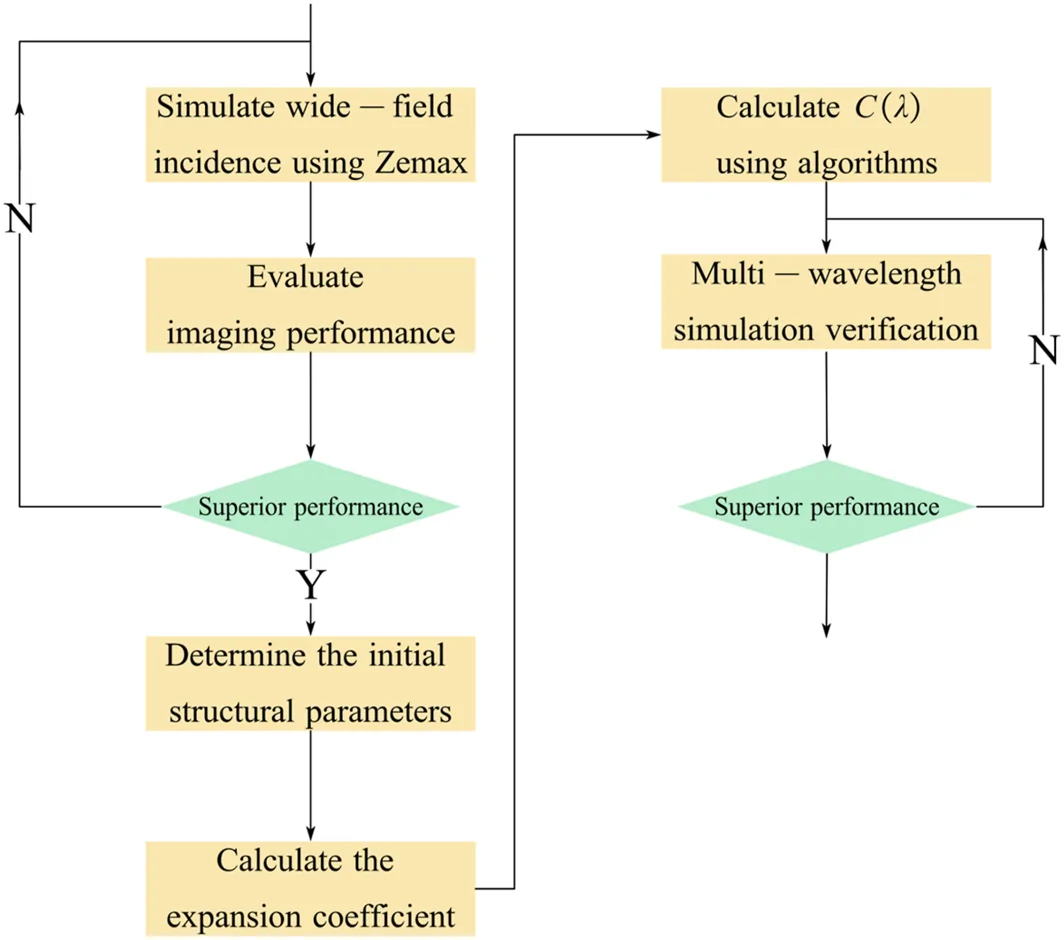
Design flowchart. Require phased iterative optimization with performance evaluation at each iteration to select the optimal solution.

## 3 Results

We optimize the physical structure parameters of the lens: the aperture diameter $R_{0}$ is 0.8 mm, the metalens lens substrate thickness d is 3 mm, the radius r is 2.292 mm, and the preset focal length f is 2.381 mm.

After obtaining the phase expansion coefficients at eight discrete wavelengths, we employ FMO and hybrid algorithm framework to match the scanned unit structure library. This ultimately calculates the corresponding compensation constant C(λ), yielding the matching phase image shown in Figure 5 (only a 160 μm segment is displayed).

**FIGURE 5:**
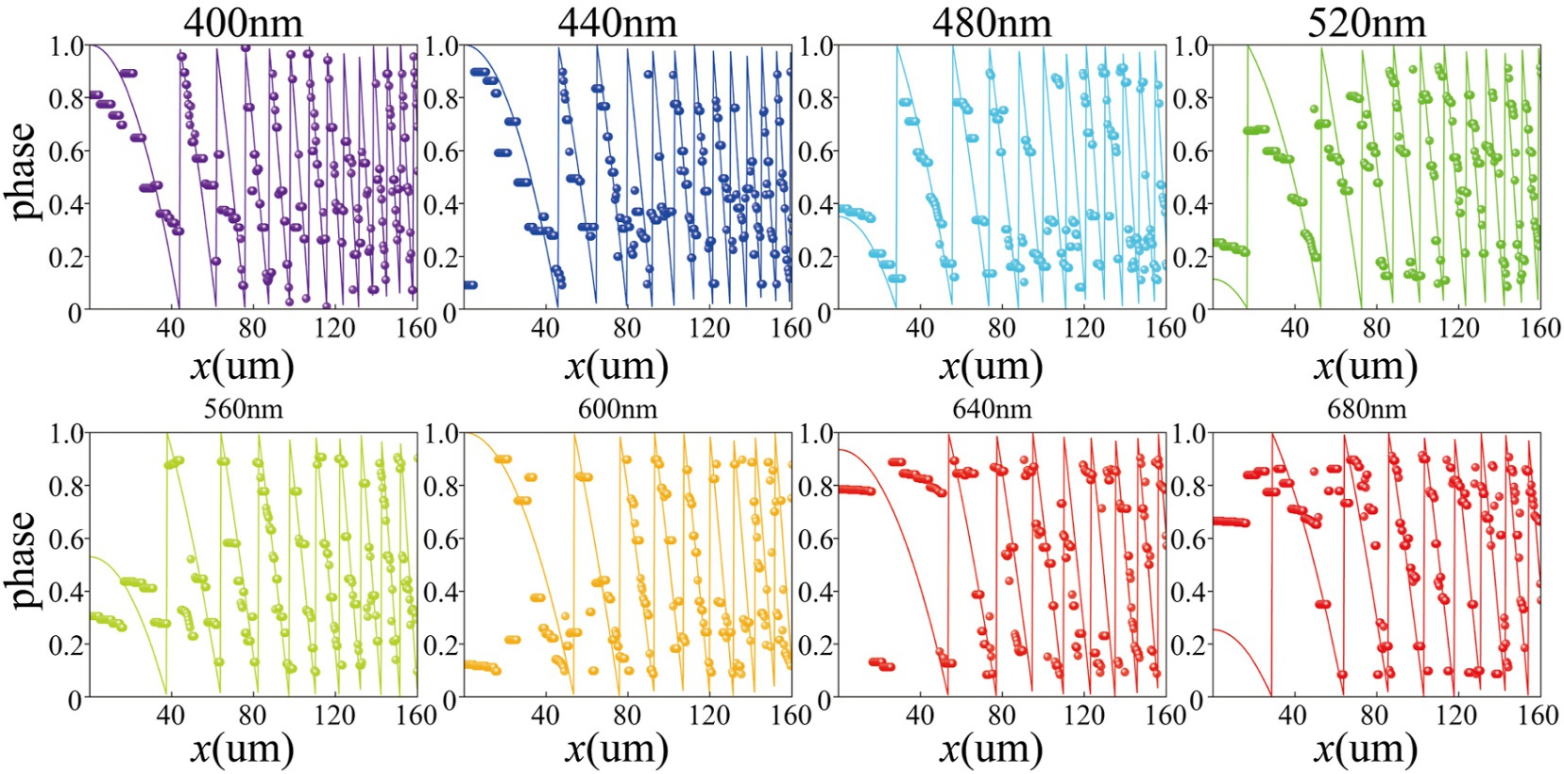
The phase matched image of the metalens at eight discrete wavelengths. When plotting, a one‐dimensional phase map is selected at y = 0 and x ranging from 0 to 160 μm. Solid lines represent the theoretical phase, while spheres denote phase compensation introduced by the actual structure. All phases have undergone normalization processing.

It should be noted that the metalens surface we designed comprises over 10^8^ unit cells. Using conventional computational methods would require a cluster of ultrahigh performance computers equipped with massive computational memory. However, with our novel approach, we complete these calculations using only a standard computer. The computational device parameters are as follows: CPU: AMD Ryzen 5 5600H with 6 cores and 12 logical processors; RAM: 16 GB at 3200 MT/s; GPU: NVIDIA GeForce RTX 3050 with 12 GB total memory. The final computation time is approximately 4 h, yielding 7.9 GB of data in *pkl* format.

It should be noted that when interpreting the plot results in Figure 5, the effects of data winding must be taken into account. Since the obtained phase data exhibits periodicity, it must be restored to its original, continuous, and uninterrupted state. On this basis, we can observe that the phase matching results are excellent.

To evaluate the mathematical robustness of the proposed FMO framework, we quantify the phase deviation resulting from mapping *N* ^2^ Cartesian grid points to a set of *N* reference radii. We calculate the exact theoretical phase for each sampling point across the entire metalens and compare it with the mapped phase assigned by the FMO algorithm. The statistical results indicate that the largest phase errors occur at the edges of the lens. The root‐mean‐square (RMS) phase error on the metalens is less than 0.53 rad, and the mean error is extremely small and negligible. The specific results are shown in Supporting Information S1: Figure S4. The Strehl ratio, calculated using the RMS, is maintained between 0.756 and 0.870, indicating good imaging performance. See Supporting Information S1: Table S2 for specific data. Similar to the discretization effects inherent in Fresnel zone plates, these deterministic high‐frequency phase perturbations primarily scatter a portion of the incident energy into background noise, resulting in a slight decrease in absolute focusing efficiency. However, this method preserves the macroscopic phase gradient and the primary focal plane geometry, achieving controllable spatial quantization error, thereby enabling the design of metalenses comprising over 10^8^ elements.

Traditional commercial FDTD software cannot perform simulations for large apertures. Therefore, to validate the performance of our designed metalens, we employ a band‐limited angular spectrum algorithm for far field diffraction calculations [46]. The band‐limited angular spectrum method is an advanced algorithm developed to address the inherent flaws (aliasing errors) of traditional angular spectrum methods in discrete numerical computations. By introducing a bandwidth‐limited transfer function, it enhances the numerical accuracy and reliability of calculations. Since full lens simulation is unfeasible, optimizing near field data acquisition is essential. We assemble near field data for the entire metalens by stitching together electromagnetic field data from the optimal unit structures matched at all grid points. This data is then used for far field diffraction calculations.

Taking into account the coupling effects between adjacent unit cells [47], we conduct simulation experiments on multiple regions (20 × 20 regions) of the metalens. The results are shown in Figure 6c. Due to the fisheye structure, the effective numerical aperture of the metalens is only about 0.17, and at specific incident angles, only specific surface regions are active; furthermore, the selected glass substrate has a refractive index of approximately 1.44, so light incident at 50° in air exhibits only about 33° in the substrate, further mitigating the effects of edge phase jumps. Although localized high‐frequency phase fluctuations and near‐field crosstalk effects still exist, the macroscopic phase gradient (i.e., the spacing and orientation of the phase stripes) exhibits excellent consistency with the ideal combined wavefront. Therefore, the effects of coupling between elements on the far‐field simulation calculations are negligible. Specific results are presented in Supporting Information S1: Section S6.

**FIGURE 6:**
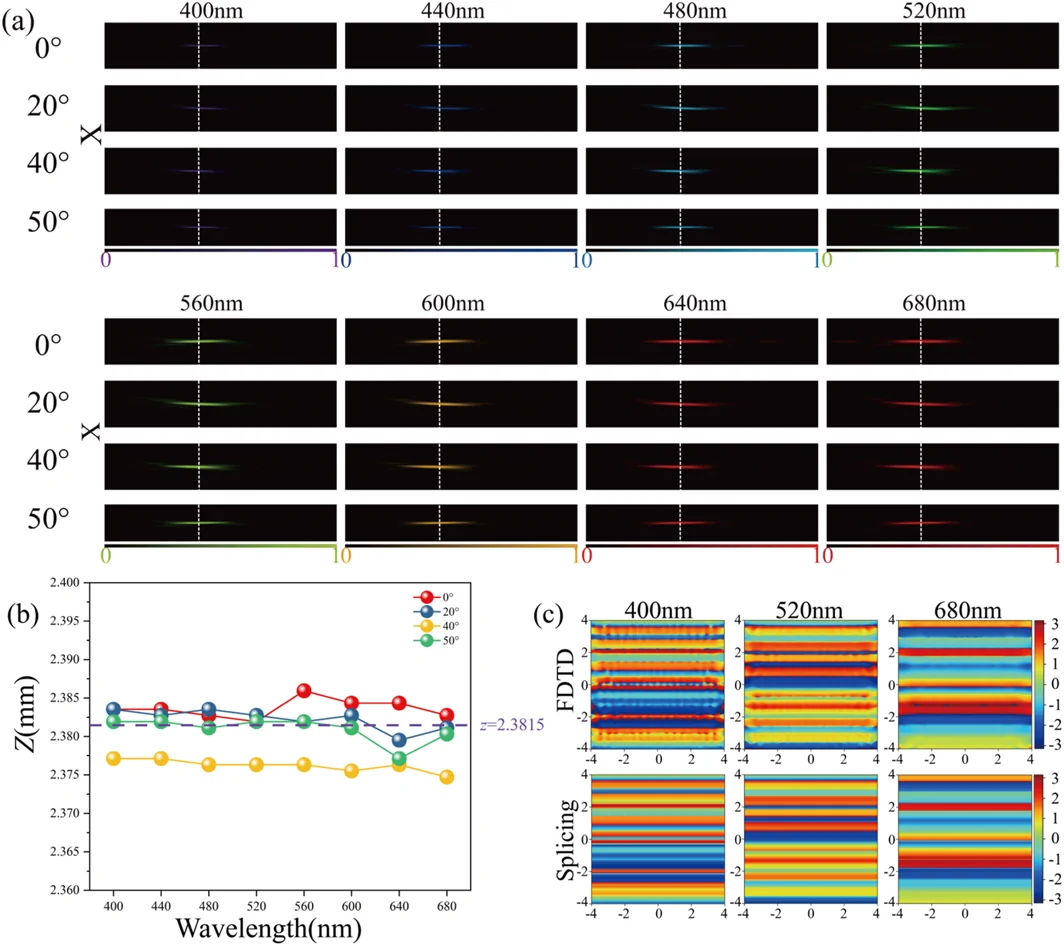
Focusing of metalens in the xz plane and analysis of coupling effects. (a) Focused images of light rays at eight different wavelengths and incident angles, white dashed lines indicate theoretical focal lengths; all data are normalized. (b) Visualized comparison of focal lengths under different incident conditions. (c) Comparison of FDTD simulations and near‐field phase stitching for a 20 × 20‐pixel region at the edge under maximum‐angle incidence at three wavelengths.

To verify the achromatic properties of the metalens across its continuous bandwidth, we perform calculations for multiple wavelengths, ensuring consistently excellent performance throughout the entire operational bandwidth. Figure 6a demonstrates that light at different wavelengths can be focused onto the same focal plane. Figure 6b provides a more detailed analysis, revealing that not only can achromaticity be achieved for the same incident angle, but also excellent achromaticity is maintained across the entire wide field of view. At an incident angle of 40°, the overall focal length exhibits the greatest deviation from the preset focal length. This occurs because the algorithm cannot guarantee optimal performance for every individual node during optimization; instead, it seeks overall optimization. At this point, the maximum deviation is only 0.284%, demonstrating excellent achromatic capability. Furthermore, the focal lengths of light with varying incident angles and wavelengths remain near the preset focal length. The minimal deviation can be fully compensated by the long depth of field, rendering its impact on imaging negligible and enhancing stability in practical applications.

We then evaluate the imaging performance of the metalens, with the results presented in Figure 7. Figure 7a shows the focused spot on the focal plane, exhibiting a nearly circular shape with minimal distortion. This indicates that the metalens delivers high imaging quality under broadband, WFOV illumination. Figure 7b displays the offset distance of the focal spot center on the focal plane relative to the theoretical origin for different incident angles. It can be observed that the spot exhibits some shift at the same incident angle but different wavelengths. However, the overall offset primarily depends on the dispersion of the substrate, and this error can be reduced by decreasing the substrate thickness. Figure 7c displays the full width at half maximum (FWHM) calculated along the y axis. Its numerical distribution approaches the diffraction limit, demonstrating excellent performance.

**FIGURE 7:**
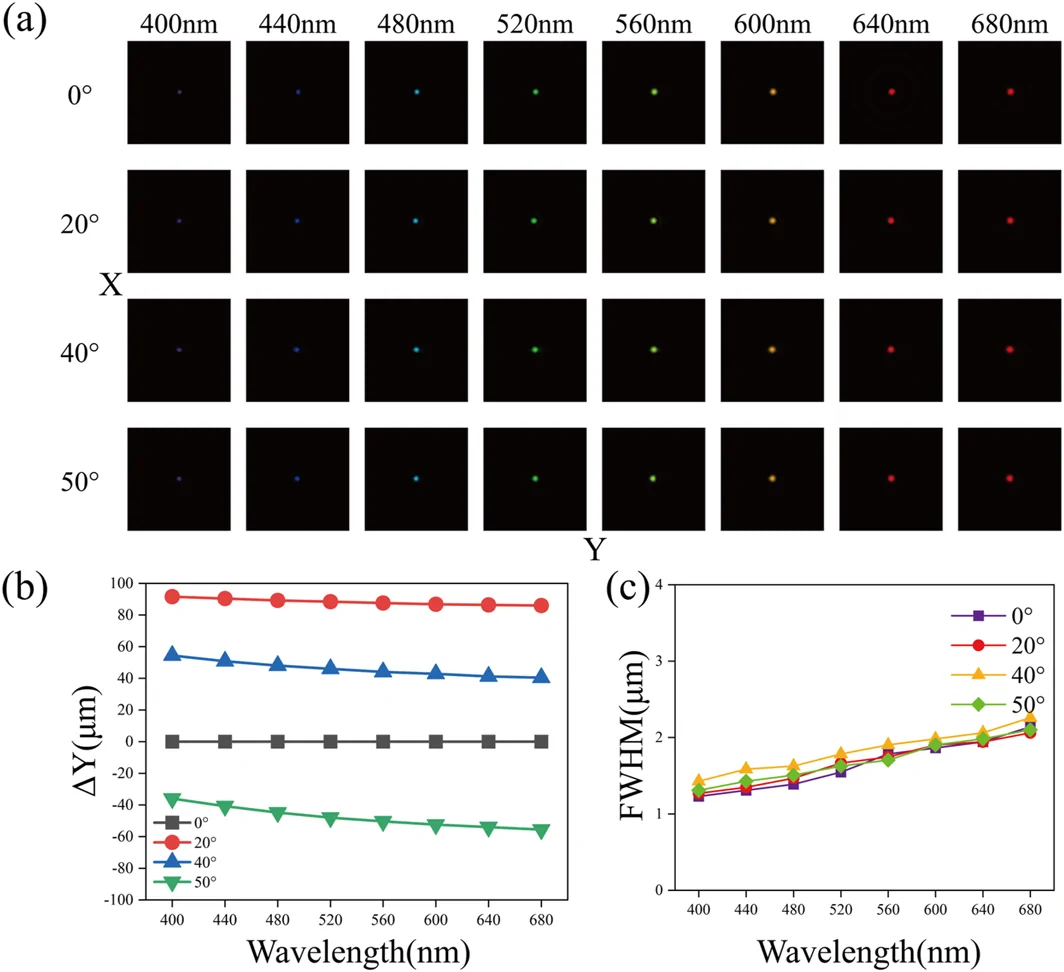
Focusing behavior and imaging performance of metalens in the xy plane. (a) Focused images of light rays at the focal plane under eight different incident angles and wavelengths, with all data normalized. (b) Displacement distance of the focal spot center relative to the theoretical origin. (c) Full width at half maximum (FWHM) calculated along the y axis.

Subsequently, we calculate the MTF curve based on the system's point spread function (PSF), as shown in Figure 8a. It can be observed that the imaging capability of the metalens approaches the diffraction limit. Similarly, we know that the imaging process of an ideal object by a lens system can be mathematically approximated as the convolution of the object's ideal geometric optical image with the system's point spread function. We perform ideal imaging processing on the metalens system using an ideal optical plane image, ensuring that the far edge of the image represents the maximum incident angle (50°). The results are shown in Figure 8b,c.

**FIGURE 8:**
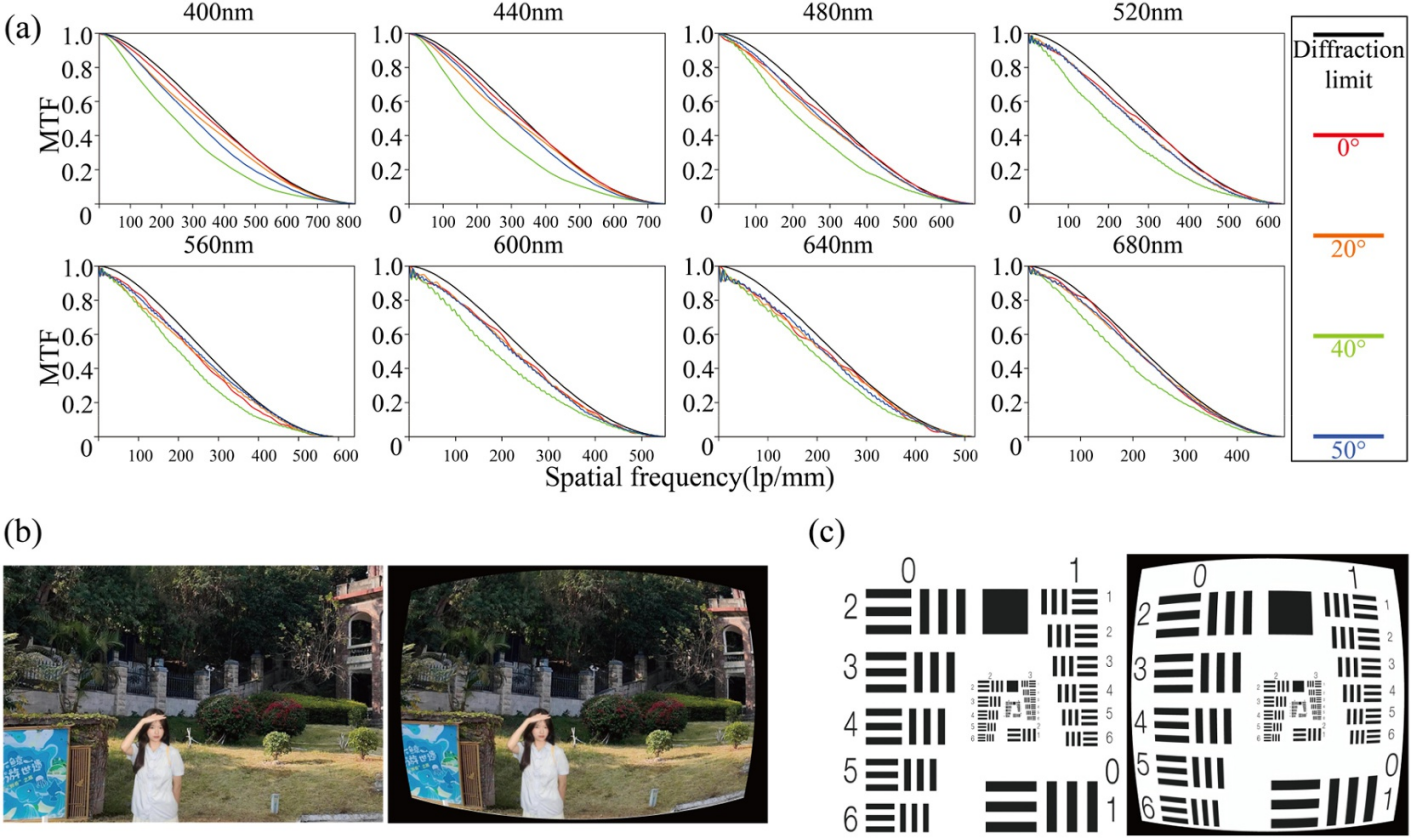
Evaluation of metalens imaging performance. (a) Tangential MTF curves calculated from PSF at different wavelengths and incident angles. (b) Real world scene image containing linear buildings, figures, and multicolored backgrounds; left shows the original image, right shows the simulated result. (c) 1951 USAF resolution target (levels 0, 1, 2, and 3). Left: original image; Right: simulated result.

As can be seen, our metalens maintains clear imaging capability even within a wide field of view spanning 50° on one side. Slight blurring appears at the image edges, primarily due to the aperture's inherent thickness, which causes information loss and undersampling for some light signals. When superimposing light of different wavelengths, the use of discrete data results in partial loss of light field information. However, employing continuous light during actual illumination will not affect the true imaging performance.

To comprehensively evaluate the optical performance of the proposed metalens, we systematically calculate its theoretical transmission efficiency and absolute focusing efficiency across the entire broadband spectrum (400–680 nm) and the target field of view (−50° to 50°). The results are presented in the form of multidimensional heatmaps in Figure 9. Figure 9a shows the calculated transmission efficiency. This transmission efficiency exhibits significant wavelength dependence: it is approximately 40.60% at a wavelength of 400 nm and reaches 84.76% at a wavelength of 680 nm, while maintaining excellent stability across different incident angles. Figure 9b presents the calculated absolute focusing efficiency, defined as the ratio of the power enclosed within the 3 × FWHM focal spot to the total incident power. The focusing efficiency peaks at 19.59% for vertical incidence at a wavelength of 680 nm and gradually decreases toward shorter wavelengths and larger incident angles.

**FIGURE 9:**
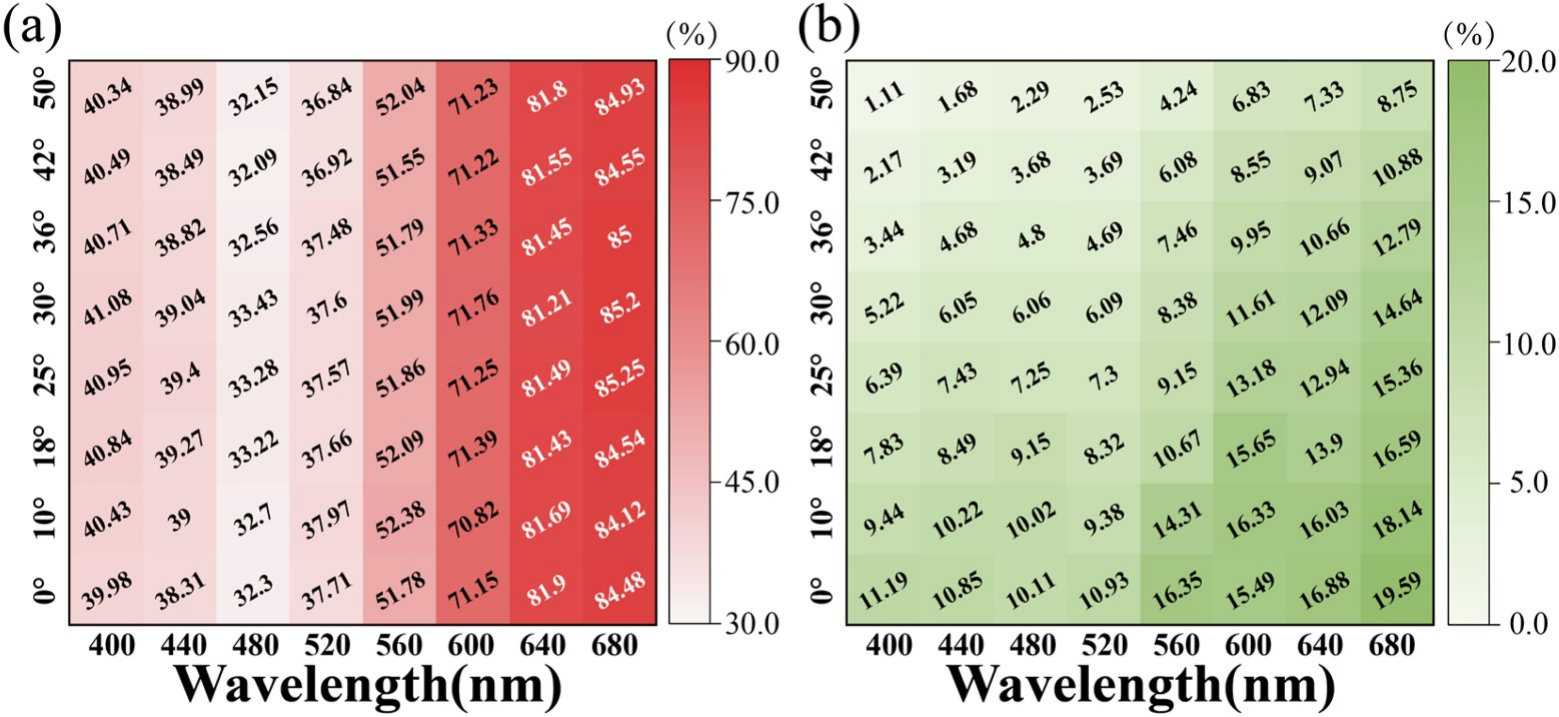
Transmittance and absolute focusing efficiency at 8 wavelengths and 8 incident angles. (a) Transmittance. (b) Absolute focusing efficiency.

This efficiency limit is in excellent agreement with the fundamental physical limit of a single‐layer broadband achromatic metalens. At shorter wavelengths, the fixed lattice period (*p* = 400 nm) approaches the limit of the Nyquist sampling criterion; at this point, strict phase requirements lead to localized near‐field scattering and high‐order diffraction losses. Conversely, at longer wavelengths (*λ* = 680 nm), strict subwavelength conditions allow for smoother phase quantization and more effective energy confinement. Furthermore, the efficiency drop observed at large incident angles is an expected result caused by high‐frequency spatial mapping errors introduced by the FMO algorithm under extreme phase gradients. The proposed efficiency metric reflects a trade‐off in strategic engineering. To address the issue of excessively dark edge imaging caused by low focusing efficiency, the weighting for wide‐angle incidence can be increased during the earlier Zemax optimization steps, resulting in a more uniform distribution of focusing efficiency values between the center and the edges. Additionally, consider incorporating a phase‐correcting metalens with a low aspect ratio and high transmittance following this metalens; this can significantly mitigate efficiency loss in wide‐field‐of‐view applications, making the design more suitable for practical use.

From the results above, it can be seen that the metalens we designed not only possesses a large field of view approaching 100° but also achieves clear achromatic imaging. Compared to traditional fisheye systems, the fisheye lens system composed of metalens features a simpler structural design. Furthermore, the design method we propose is universal; it significantly reduces computational requirements while improving processing speed and accuracy. In Supporting Information S1: Section S7, we also employ the method to achieve achromatic correction for vortex beams. Unlike the simulations in this study, the phase distribution on the surface of the vortex‐light metalens does not exhibit rotational symmetry. By employing FMO method—constructing an approximate set mapping of different Fresnel zones and mapping each topological region's grid cell in each ring band to a single neighboring grid parameter—we reduce the computational complexity from two dimensions to one. The simulation results also indicate that, specifically for large‐aperture achromatic correction problems, our method remains applicable even for vortex beams where the surface phase distribution is not ring‐symmetric.

In addition, we have included test results at 10° and 30° incident angles in Supporting Information S1: Section S8 to ensure performance stability in the intermediate angle transition region.

Although traditional manufacturing processes currently face challenges due to extreme parameters such as 100‐nm pitch and massive cell counts, our FMO framework demonstrates exceptional flexibility. It can easily meet minimum feature size (MFS) constraints, making it compatible with state‐of‐the‐art scalable manufacturing technologies, such as roll‐to‐roll nanoimprint lithography [48]. Given current processing technologies, for a 4.584 mm aperture and a 400 nm period, proximity effects must be accounted for in electron beam lithography (EBL) or DUV step‐and‐repeat exposure processes. Our FMO method ingeniously simplifies the implementation of proximity effect correction algorithms during the GDS mask generation stage. By using FMO to preprocess the distribution density of surface unit structures, local dose compensation techniques can be applied to mitigate pattern distortion. Similarly, since FMO requires the surface unit mapping to be determined in advance, this means that the required types of unit structures can be determined in advance, that is, the surface phase partitioning is determined in advance. This is highly compatible with calling functions from the C++‐based gdstk library, enabling GDS layout generation time to be reduced by more than two orders of magnitude.

## 4 Conclusion

We have developed an entirely new metalens design methodology that unifies the large aperture, wide field of view, and achromatic capabilities of metalens operating in the visible light spectrum. This approach significantly reduces computational complexity, enabling the design of large aperture metalens with over 10^8^ elements without relying on large supercomputing clusters.

Our approach is a design methodology that mimics the structure of traditional fisheye lenses. It directs incident light from different angles through an aperture system onto distinct unit cells within the metalens. Zemax optimization corrects wide field aberrations, while a hybrid algorithm framework and FMO process the massive data space to screen and arrange unit structures. This ultimately achieves chromatic aberration elimination across different wavelengths.

In our numerical demonstration, we design and implement a metalens with a large aperture of 4.584 mm, a field of view approaching 100°, and operating between 400–680 nm with excellent focusing characteristics. By integrating three mutually constrained parameters onto a single metalens lens, we reduce computational complexity from O(*N* ^2^) to O(*N*) while achieving high imaging performance. This comprehensive design approach provides a reference for metalens development in other directions, enabling extension to different wavelength bands and more complex optical functions. It will accelerate the practical application of metalens, allowing it to play a significant role in broader fields.

## Author Contributions

**Zongyuan Chen:** conceptualization, methodology, software, investigation, formal analysis, visualization, validation, writing – original draft, writing – review and editing. **Wangzhe Zhou:** conceptualization, writing – review and editing. **Shaoqi Li:** conceptualization, writing – review and editing. **Yiyi Li:** conceptualization, writing – review and editing. **Nan Gu:** conceptualization, writing – review and editing. **Man Yuan:** conceptualization, writing – review and editing. **Junbo Yang:** conceptualization, methodology, supervision, funding acquisition, resources, project administration, writing – review and editing.

## Funding

This work was supported by the National Key Research and Development Program of China (2022YFF0706005), the National Natural Science Foundation of China (62275271, 62305387, 12272407, 62405037, and 62275269), and Chongqing Natural Science Foundation (CSTB2024NSCQ‐MSX0581, CSTB2024NSCQ‐LZX0033).

## Supporting information


Supporting Information S1

## Data Availability

The methods employed in this study and the datasets generated or analyzed may be obtained from the corresponding author upon reasonable request.
