# Supplementary material for: Large‐Aperture, WFOV Broadband Achromatic Metalens Enabled by Forward Mapping Optimization and Hybrid Algorithmic Framework
Source: Nanophotonics. 2026 Aug 6;15(15):e70228. doi: 10.1002/nap2.70228 (PMC13642539; doi:10.1002/nap2.70228)
Supplement: Supplementary file 1 [file NAP2-15-e70228-s001.docx]

Supporting Information

Large-aperture, WFOV broadband achromatic metalens enabled by forward mapping optimization and hybrid algorithmic framework

Zongyuan Chen, Wangzhe Zhou, Shaoqi Li, Yiyi Li, Nan Gu, Man Yuan, and Junbo Yang*

Z. Y. Chen, W. Z. Zhou, S. Q. Li, Y. Y. Li, N. Gu, M. Yuan, and J. B. Yang

College of Science, National University of Defense Technology, Changsha 410073, China.
E-mail: yangjunbo@nudt.edu.cn

**S1. Hybrid algorithm optimization framework**

Our hybrid algorithm framework supports various combinations of algorithms. To verify its effectiveness, we evaluated three algorithms—particle swarm optimization, differential evolution, and genetic algorithms—as post-processing algorithms following Bayesian optimization, and plotted their convergence curves as shown in **Figure S1**.

**Figure S1.** Convergence curves of different algorithms.

The common parameter settings of several algorithms are all kept consistent (the initial population size is set to 150, the number of iterations is 500, and the upper and lower bounds of variables are the same), in order to control the influence of the differences in initial values of the algorithms. The final iterative results are almost consistent, which can rule out the validity of specific algorithms, indicating that this hybrid algorithm framework has algorithmic universality.

**S2. Scanning results**

In the optical simulation software FDTD, the scan parameters are set to vary both the side length and diameter from 100 to 350 nm with a step size of 0.5 nm.

**Figure S2.** Results obtained from unit cell structure scanning. (a) Simulation results of rectangular prism phase as a function of wavelength and cross sectional edge length; (b) Simulation results of cylindrical phase as a function of wavelength and cross sectional diameter; (c) Simulation results of rectangular prism transmittance as a function of wavelength and cross sectional edge length; (d) Simulation results of cylindrical transmittance as a function of wavelength and cross sectional diameter.

**S3. Coefficients**

**Table S1.** The eight sets of coefficients obtained through Zemax iterative optimization.

| Wavelengths [nm] | *A*_1_ | *A*_2_ | *A*_3_ | *A*_4_ | *A*_5_ | *A*_6_ |
| --- | --- | --- | --- | --- | --- | --- |
| 400 | -3272.442 | 7.744 | -5.017 | 1.363 | -0.182 | 0.010 |
| 440 | -2974.962 | 6.209 | -3.921 | 1.013 | -0.129 | 0.007 |
| 480 | -2727.061 | 5.020 | -3.088 | 0.751 | -0.089 | 0.005 |
| 520 | -2517.298 | 4.073 | -2.434 | 0.549 | -0.059 | 0.003 |
| 560 | -2337.501 | 3.302 | -1.909 | 0.389 | -0.035 | 0.001 |
| 600 | -2181.675 | 2.665 | -1.479 | 0.259 | -0.016 | 0.000 |
| 640 | -2045.328 | 2.130 | -1.120 | 0.151 | -0.001 | -0.001 |
| 680 | -1925.020 | 1.677 | -0.818 | 0.060 | 0.013 | -0.001 |

**S4. Verification simulation on a small aperture metalens**

The aperture diameter$\text{ }\text{R}_{\text{0}}\text{ }$was set to 24 μm, the substrate thickness $\text{d}$ to 90 μm, the focal length $\text{f}$ to 71.444 μm, and the metalens radius $\text{r}$ to 68.764 μm.

**Figure S3.** Simulation verification of a small aperture metalens. (a) Schematic diagram of the metalens structure; (b) Phase surface obtained from FDTD simulation; (c) Focusing behavior at three wavelengths under normal incidence.

**Figure S3**(b) shows the phase distribution of three wavelengths transmitted through the aperture of the metalens under normal incidence, exhibiting excellent focusing characteristics. Figure S3(c) illustrates the focusing behavior at the focal plane for the three wavelengths under normal incidence. Here, significant interference light is observed, primarily due to the small aperture size causing pronounced resonance effects between unit cells and between the cells and the aperture. Nevertheless, the focal spot exhibits a predominantly circular shape with concentrated energy distribution, indicating the effectiveness of the employed method. Subsequent experiments with larger apertures demonstrate that this interference term is substantially reduced.

**S5. Global root-mean-square (RMS) phase error**

We compare the phase obtained after matching using the FMO method with the theoretical phase, and calculated the root-mean-square (RMS) error and the mean error, as shown in **Figure S4**. As can be seen, the mean error is extremely small, indicating that the surface phase is, on the whole, very close to the theoretical value. According to the Maréchal criterion, when the RMS wavefront error is ≤ *λ*/14, the system can be considered to be near the diffraction limit.^[1]^ We calculate the Strehl ratio using **Equation S1**, as shown in **Table S2**.

$S\approx e^{-(\frac{2\text{π}}{\lambda}\sigma)^{2}}=e^{-\omega^{2}}$ (S1)

Where $\text{σ}$ is the RMS error, and $\text{ω}$ is its radian representation.

**Table S2.** The Strehl ratio calculated at 8 wavelengths.

|  | 400nm | 440nm | 480nm | 520nm | 560nm | 600nm | 640nm | 680nm |
| --- | --- | --- | --- | --- | --- | --- | --- | --- |
| Strehl ratio | 0.8695 | 0.8497 | 0.7844 | 0.8080 | 0.8017 | 0.7735 | 0.7557 | 0.7698 |

As can be seen, at the selected wavelength, the Strehl ratio is close to or greater than 0.8, indicating good performance.

**Figure S4.** Root mean square error and mean error.

**S6. Analysis of unit coupling effects**

To investigate the effects of near-field coupling, we select five small regions on the metalens surface, each consisting of 20×20 units, corresponding to fields of view of 0.7 mm, 1.1 mm, 1.5 mm, and 1.9 mm, as well as a maximum field of view of 50° (as shown in Figure 6(c)). First, we compare the near-field phase data at wavelengths of 400 nm, 520 nm, and 680 nm, as shown in **Figure S5**(a)–(d) (corresponding to 0.7 mm, 1.1 mm, 1.5 mm, and 1.9 mm fields of view, respectively). It can be seen that the coupled phase distribution is consistent with the phase distribution obtained from stitching. To quantitatively calculate the error, we compute the root mean square (RMS) error and estimated its impact on the Strehl ratio; the results are shown in **Table S3**.

**Figure S5.** Under different incident conditions, the FDTD simulation results for a 20×20-pixel region at the edge were compared with the near-field phase stitching results at three wavelengths. (a) 0.7 mm field of view. (b) 1.1 mm field of view. (c) 1.5 mm field of view. (d) 1.9 mm field of view.

**Table S3.** RMS and Strehl ratio errors obtained for different fields of view at three wavelengths (the coupling results divided by the stitching results are shown in parentheses).

|  | 0.70mm | 1.10mm | 1.50mm | 1.90mm | 2.29mm(50°) |
| --- | --- | --- | --- | --- | --- |
| 400nm | 0.472rad (0.800) | 0.430rad (0.831) | 0.617rad (0.683) | 0.598rad (0.699) | 0.475rad (0.798) |
| 520nm | 0.239rad (0.945) | 0.413rad (0.843) | 0.472rad (0.800) | 0.326rad (0.899) | 0.328rad (0.898) |
| 680nm | 0.3646rad (0.876) | 0.306rad (0.911) | 0.492rad (0.785) | 0.283rad (0.923) | 0.218rad (0.954) |

To account for the effects of the focusing effect on the central spot, we perform far-field diffraction calculations for small regions with large Strehl ratio errors, then back-calculated their diffraction into the central field to obtain a one-dimensional expansion of the light field, as shown in **Figure S6**.

**Figure S6.** One-dimensional plot of the diffracted light field at the center.

Comparison results show that the actual far-field peak intensity, after accounting for near-field coupling effects, is lower than that of the ideal stitching model, and this attenuation effect is more pronounced in the short-wavelength range. However, quantitative calculations indicate that the average Strehl ratio, despite phase perturbations in the near field, remains above 0.8, satisfying the imaging criteria for diffraction-limited resolution. At the same time, Figure S6 shows that near-field coupling does not cause significant distortion in the spatial energy distribution of the central main lobe; its primary negative effect is that it scatters some of the energy into the high-frequency background, thereby slightly reducing the total effective received light energy and the final imaging intensity, and is therefore negligible.

**S7. Large-aperture vortex achromatic metalens**

To verify the scalability of our proposed method, we simulate a large-aperture vortex achromatic metalens operating in the 950–1100 nm wavelength band. The structural unit has a period of 400 nm, a manufacturing tolerance of 120 nm, and a height of 1000 nm; we select a structure with a polarization conversion efficiency greater than 0.8. The metalens has an aperture of 1.2 mm, a focal length of 30 mm, a Fresnel zone number of 4, and a topological charge increment of 1.

The method for correcting chromatic aberration relies on a combination of transmission phase and geometric phase, as shown in **Equation S2**.

 (S2)

The transmission phase compensates for the phase difference caused by different wavelengths (the method remains consistent with the main text); the geometric phase compensates for the remaining error, including the helical phase, as shown in **Equation S3**.

(S3)

Using the FMO method, each ring-band element is mapped to a single mesh parameter in the adjacent topological region; consequently, the mesh elements of the two-dimensional surface are also mapped to a one-dimensional single ring. Using the same hybrid optimization framework, the results ultimately demonstrate only single-wavelength focusing and achromatic effects, as shown in **Figure S7**.

**Figure S7.** Spiral-shaped achromatic correction (intensities normalized). (a) 0.85x focal length. (b) 0.90x focal length. (c) 1.00x focal length. (d) 1.10x focal length. (e) 1.15x focal length. (f) 3D view and projections in the *xy* direction. (g) Intensity in the *xz* plane at wavelengths of 950, 1000, and 1064 nm.

Figure S7(a)–(e) shows the focused spot in the *xy* plane at different distances under incident light at a wavelength of 950 nm. It can be seen that the spot continuously rotates with increasing depth, consistent with the characteristics of a single-helix vortex beam. Figure S7(f) illustrates the beam propagation characteristics from a 3D perspective and their projections in the *xy* direction. (For ease of observation, we have selected only the portions with an intensity greater than 90% in each *xy* cross-section.) The full-wavelength achromatic correction results are shown in Figure S7(g), with wavelengths from left to right being 950 nm, 1000 nm, and 1064 nm. The maximum focal length error is controlled within 3.4%, meeting the criteria for good achromatic correction. In summary, our FMO method can also be applied to perform achromatic optimization of vortex beams. By making corresponding adjustments to the set mapping while keeping other conditions constant, we demonstrate that our proposed method possesses excellent generalizability.

**S8. Supplementary Information for 10° and 30° Incidence Angles**

To more comprehensively evaluate the performance of the metamaterial lens as it varies continuously—particularly its performance in the intermediate angle transition region—we have included results for 10° and 30° incident angles.

**Figure S8**–**S13** show, respectively, the *xz*-plane focusing analysis of the metalens under two incident angles, the variation in focal length, the *xy*-plane focusing analysis, the displacement of the focal center relative to the theoretical origin, the full width at half maximum, and the tangential MTF curve calculated based on the point spread function.

**Figure S8.** The *xz*-plane focusing analysis of the metalens under two incident angles.

**Figure S9.** Changes in the focal length of a metalens at two different angles of incidence.

**Figure S10.** Analysis of focusing in the *xy* plane for the metalens at two incident angles.

**Figure S11.** The displacement of the focal center of the metalens relative to the theoretical origin at two different incident angles.

**Figure S12.** The full width at half maximum of the metalens at two incident angles.

**Figure S13.** Tangential MTF curves for a metalens calculated using the point spread function at two incident angles.

**References**

1.	Mahajan, Virendra N. “Strehl ratio for primary aberrations in terms of their aberration variance.” *Journal of the Optical Society of America* (1983) 73(6): 860-861.
